# Integration of Adjunctive Therapy for Congenital Adrenal Hyperplasia

**DOI:** 10.3390/children12070898

**Published:** 2025-07-08

**Authors:** Phyllis W. Speiser

**Affiliations:** 1 Department of Pediatrics, Donald and Barbara Zucker School of Medicine at Hofstra–Northwell, Hempstead, NY 11549, USA; pspeiser@northwell.edu; 2 Cohen Children’s Medical Center of New York, New Hyde Park, NY 11040, USA

## 1. Introduction

CAH represents a prototypical enzyme deficiency disorder, most commonly affecting steroid 21-hydroxylase, in which the critical adrenal pathway from cholesterol to cortisol is blocked. The lack of the important end-product, cortisol, the human flight-or-fight hormone, stimulates the HPA axis. Hypothalamic CRF1 activates adrenocorticotropic hormone (ACTH), which in turn drives a futile cycle of attempted cortisol production, instead causing accumulation of precursors shunted to sex hormones. This process begins during the mid-first trimester of prenatal life when adrenal steroid secretion is initially observed [1]. Hence, classically affected females are exposed to testosterone in utero resulting in masculinization of their external genitalia. This phenotype may be recognized as a diagnostic clue in newborns; however, males show no obvious stigmata. In about 75% of affected individuals, secretion of the equally important mineralocorticoid, aldosterone, is insufficient. Systemic salt and fluid loss, hypotension, shock and death may ensue if untreated, the dreaded “adrenal crisis”. For these reasons, infant mortality from CAH was estimated at about 4% prior to the advent of newborn screening [2]. Prompt diagnosis and initiation of hydrocortisone treatment prevents further virilization; additional treatment with a mineralocorticoid analog, fludrocortisone, and sodium chloride supplements helps maintain sodium and fluid balance.

Clinicians have long realized that two or more daily oral doses of hydrocortisone cannot precisely mimic the physiologic circadian rhythms of the HPA axis. Consequently, patients are, by turn, often either under-treated or over-treated. Adverse side effects of insufficient glucocorticoid treatment include sex hormone-induced premature adrenarche, early epiphyseal maturation with advanced bone age seen on radiographic imaging, statural overgrowth in early childhood with adult stature below that expected for parental heights, and disruption of the hypothalamic–pituitary–gonadal axis with central precocious puberty and reproductive dysfunction in early adult life. Cessation of glucocorticoid treatment or failure to recognize the need for increased dosing during illness or major surgery may lead to adrenal crisis and even death. Conversely, adverse effects of glucocorticoid over-treatment include excessive weight gain, stunting of statural growth, elevated blood pressure, metabolic syndrome, lower bone mineral density and poor quality of life. Since treatment must be maintained life-long, this balancing act is challenging and consensus treatment guidelines must be individualized according to growth parameters and priorities at various life stages.  

## 2. Discussion

Healthy children secrete cortisol at a mean rate of approximately 6 ± 2 mg/m^2^/day [3,4]. Consensus guidelines for oral hydrocortisone replacement suggest treatment equivalent to 10–15 mg/m^2^/day, ideally given in three divided doses in children affected with CAH [5]. The rationale for a supra-physiologic CAH replacement regimen is that HPA overdrive is difficult to suppress. Cortisol replacement is not the sole goal of therapy; rather to control abnormal androgen secretion higher hydrocortisone doses are required. However, too much hydrocortisone is potentially toxic; published data indicate that adolescent growth may be suppressed above 17 mg/m^2^/day [6]. Thus, one ought to prescribe the lowest total daily dose of hydrocortisone that can achieve adequate control, but what constitutes adequate control is elusive and rather arbitrarily defined, especially in growing children.

To address these confounding problems in CAH management, several new drug formulations have been developed. Until recently many countries had access to only 10 mg hydrocortisone tablets, an unsuitably large dose for infants and children even when crushed and compounded in smaller amounts, a process sometimes fraught with errors [7,8]. Immediate-release oral hydrocortisone granules have been made available in the US (Alkindi Sprinkle, Eton Pharmaceuticals, Deer Park, IL, USA) and Europe (Neurocrine UK, Cardiff, UK) in units of 0.5, 1, 2 and 5 mg, allowing safe and appropriate dosing to infants and toddlers [9,10]. For adolescents age 12 and older and adults with CAH, a modified release formulation of hydrocortisone (MRHC) has been in use in Europe. This preparation is given in two oral doses, 1/3 in the morning and 2/3 at bedtime, aiming in particular to suppress the early morning surge in adrenal androgen. An open label randomized phase 3 study in adults that compared modified release hydrocortisone with equivalent doses of prednisolone demonstrated lower total daily dosing, closer simulation of circadian rhythm, and better adrenal control with the former medication [11]. Although no systematic studies have been performed in adolescents, European experts have suggested dosing guidelines for MRHC in young CAH patients [12]. An additional variation on the theme of hydrocortisone replacement is continuous subcutaneous hydrocortisone infusion (CSHI), adapted from insulin-dependent diabetes treatment technology. A phase 2 trial in a small number of adults with uncontrolled CAH showed improved androgen control, lean body mass and quality of life, along with modest reductions in total daily hydrocortisone dosing [13]. These newer therapeutic options have not gained wide acceptance as yet, in part due to logistical and cost challenges.  

Moving beyond variations in methods for glucocorticoid replacement are CRF1 receptor antagonists. Of two such drugs, one has advanced past phase 3 pediatric and adult clinical trials and received fast-track approval from the US Food and Drug Administration in late 2024. Crinecerfont (Crinessity, Neurocrine Biosciences, San Diego, CA, USA) was tested versus placebo in prospective, randomized trials while study subjects were maintained on standard treatment with glucocorticoids (hydrocortisone or predniso(lo)ne) and mineralocorticoids. Both primary and secondary endpoints were achieved. In the pediatric trial, 100 of 103 subjects were retained for the full 28 weeks. Among 69 participants receiving crinecerfont, mean morning serum androstenedione levels were substantially reduced at 4 weeks (*p* < 0.001 vs. 34 in the placebo group) reflecting improved androgen control. Moreover, the mean glucocorticoid dose decreased by 18% at 28 weeks while androstenedione control was maintained in the crinecerfont group. In contrast, placebo recipients increased their glucocorticoid doses by 5.6% (*p* < 0.001) [14]. Similarly, the larger adult trial (*n* = 182) also showed 97% retention at week 24, and week 4 mean serum androstenedione levels decreased by 345 ng/dl (*p* < 0.001 vs. placebo group). Physiologic glucocorticoid dosing was achieved with androstenedione control in 63% of the crinecerfont group at week 24 [15]. Interestingly, only 30% of pediatric subjects achieved physiologic glucocorticoid dosing defined as ≤11 mg/m^2^/day. Adverse events, commonly headache in both children and adults, fever and vomiting in children, and fatigue in adults, did not induce subjects to discontinue treatment. Phase 4 clinical trials are being initiated to determine long-term efficacy and safety for CAH patients.  

The reason for fewer pediatric trial subjects attaining physiologic glucocorticoid dosing is unclear. One explanation might be that achieving adrenal control during puberty presents a greater challenge compared with the relatively static body composition and hormonal balance among prepubertal children or adults; nearly half of the pediatric crinecerfont group were assessed as having attained Tanner stages 2 to 4, i.e., active puberty. Additionally, a substantial proportion of adult subjects were treated with long-acting and more potent glucocorticoids (prednis(ol)one or dexamethasone, typically) compared with pediatric subjects most commonly treated with short-acting hydrocortisone. Potent synthetic glucocorticoids are generally avoided in children for potential growth-stunting effects. In adults, predniso(lo)ne and dexamethasone facilitate adrenal suppression, but often induce excess weight gain and undesirable cardiometabolic changes.

## 3. Conclusions

Innovations in CAH management, long overdue, are now being realized. The coming years will elucidate how best to integrate the alternative and adjunctive therapies with standard glucocorticoid regimens. Front runners in the field from present perspectives include novel hydrocortisone delivery, most prominently MRHC, and CRF1 receptor antagonists. Several obstacles, not least of which is cost, need to be addressed before these approaches are employed by clinicians and patients.

Encouraging clinical trial results suggest that new approaches to management may benefit the CAH patient population. Specifically, alternative means for hydrocortisone delivery may be useful in some patients who are uncontrolled with standard multiple daily dosing. These strategies do not necessarily exclude use of adjunctive medications, such as crinecerfont, when glucocorticoid management falls short of the therapeutic goal. One may envision the intermittent use of alternate hydrocortisone delivery and/or adjunctive medication in some patients.

### Future Directions

Questions remaining include the following: (1) How to balance the risks and benefits of novel therapies. Protocols have yet to be established for managing stress dosing in CAH patients treated with modified release hydrocortisone, nor has pediatric safety and efficacy been tested for this type of medication. (2) Can the added costs of alternative therapies be justified? For instance, in the UK, the National Health Service cost for a 5 mg Efmody modified release hydrocortisone pill is GBP 2.7, about ten-fold higher than the cost of a standard hydrocortisone 5 mg tablet. Even more striking is the US list price for Crenessity, where a 30 day supply for a typical 4–6 year old child weighing <20 kg is about USD 20,000, although some patients may be eligible for cost reduction. (3) What is the optimal age to begin novel or adjunctive treatment? Finally, data are as yet unavailable as to the long-term clinical benefits of alternative hydrocortisone preparations in the CAH population [16]. Clearly, more data are needed to provide a rationale for the prudent use of new medications.

Other novel approaches to CAH treatment are under investigation. Short-term data from a phase 2 trial for a selective MC2R (ACTH receptor) antagonist, atumelnant (CRN04894) appear promising when presented in abstract form, but are as yet unpublished [17]. The MC2R antagonist acts one step more proximal to the pituitary and adrenal glands compared with the CRF1 receptor antagonist for similar, but separate, adjunctive therapy. Over 90% of subjects experienced reductions in androstenedione and 17-hydroxyprogesterone in 12 weeks while treated once daily with oral atumelnant; phase 3 trials are poised to begin soon. Experimental medications that have not advanced beyond phase 1–2 trials include ACTH monoclonal antibodies, androgen receptor antagonists and aromatase inhibitors, steroidogenesis inhibitors, and cell therapies [reviewed in [18]].

It is unlikely that the treatments discussed here will represent standalone treatments for all patients, nor do they represent a cure for CAH. A cure would mean replacing the missing adrenal enzyme. Gene therapy, which has attained some success in animal studies [19] and in children suffering from other monogenic disorders [20], has not yet been realized in CAH patients. An early phase clinical trial in adults for an adeno-associate virus 5 vector-based gene therapy [21] has recently been discontinued due to disappointing efficacy.

## Abbreviations

congenital adrenal hyperplasia (CAH); corticotropin-releasing factor type 1 (CRF1); hypothalamic–pituitary–adrenal (HPA) axis; adrenocorticotropic hormone (ACTH); modified release formulation of hydrocortisone (MRHC); continuous subcutaneous hydrocortisone infusion (CSHI); MC2R (ACTH receptor).
